# A Mexican Honeymoon Marred by Gastrointestinal Upset: A Case of Dientamoeba fragilis Causing Post-infectious Irritable Bowel Syndrome

**DOI:** 10.7759/cureus.1992

**Published:** 2017-12-27

**Authors:** Saeed Ali, Neelam Khetpal, Muhammad Talha Khan, Mamoon Rasheed, FNU Asad-Ur-Rahman, Karen Echeverria-Beltran

**Affiliations:** 1 Internal Medicine Residency, Florida Hospital-Orlando; 2 Internal Medicine, Khyber Medical College; 3 Gastroenterology, Cleveland Clinic Florida

**Keywords:** dientamoeba fragilis, postinfectious ibs, stool ova & parasite

## Abstract

Dientamoeba fragilis (D. fragilis) is an anaerobic intestinal protozoan parasite that has been associated with irritable bowel syndrome (IBS)-like symptoms. We report a case of post-infectious IBS caused by D. fragilis treated successfully with metronidazole.

A 33-year-old African American male with an unremarkable past medical history was seen in the office with a three-month history of intermittent, generalized, crampy abdominal pain with bloating and flatulence without associated weight loss. He visited Mexico for his honeymoon four months ago. Initial lab work was normal. Dietary changes including fermentable oligosaccharides, disaccharides, monosaccharides, and polyols (FODMAP) diet and loperamide were prescribed with the presumptive diagnosis of IBS; however, his symptoms persisted. Three samples of stool for ova and parasites (O&P) were positive for D. fragilis. The patient was treated with metronidazole for 14 days. Repeat fecal O&P were negative. Upon follow-up, the patient’ symptoms substantially improved with a resolution of abdominal pain and flatulence.

Infection caused by D. fragilis may be symptomatic or asymptomatic. It is transmitted by the fecal-oral route. Symptoms include abdominal pain, bloating, and alteration of bowel movements, resembling IBS. The diagnosis is made via the detection of D. fragilis trophozoites in appropriately fixed and stained stool samples or by a polymerase chain reaction. Treatment options include tetracyclines, paromomycin, metronidazole, and Iodoquinol. Further epidemiologic studies may help in elucidating the association between D. fragilis and IBS.

## Introduction

Parasitic infections are widespread in both urban and rural areas throughout the world. Their prevalence is higher in areas of poor sanitation and unhygienic drinking water, as they are transmitted via the fecal-oral route [1].

Parasitic infections, such as Dientamoeba fragilis (D. fragilis), cause abdominal pain, bloating, and the alteration of bowel habits mimicking IBS [2-3]. D. fragilis has been reported as a cause of irritable bowel syndrome (IBS), allergic colitis, and diarrhea in human immunodeficiency virus (HIV) patients. It has been accepted as a pathogen in some countries whereas it has been overlooked and disregarded in others [3].

D. fragilis is very difficult to detect unless suitable staining or culture methods are used [3]. Diagnostic tests for D. fragilis are not routinely available in all diagnostic laboratories, and it is often underdiagnosed. Its trophozoite form usually degenerates rapidly after leaving the gut and is easily missed in direct saline or iodine preparations [4]. The most sensitive detection method is the parasite culture where the culture media require rice starch to provide a carbohydrate source [5].

Many antimicrobial agents have been used to eradicate the D. fragilis infection, including tetracyclines, paromomycin, metronidazole, and iodoquinol [6]. Therapy is needed to eradicate this organism, as studies have shown the resolution of symptoms with treatment [7].

## Case presentation

**Figure 1:**
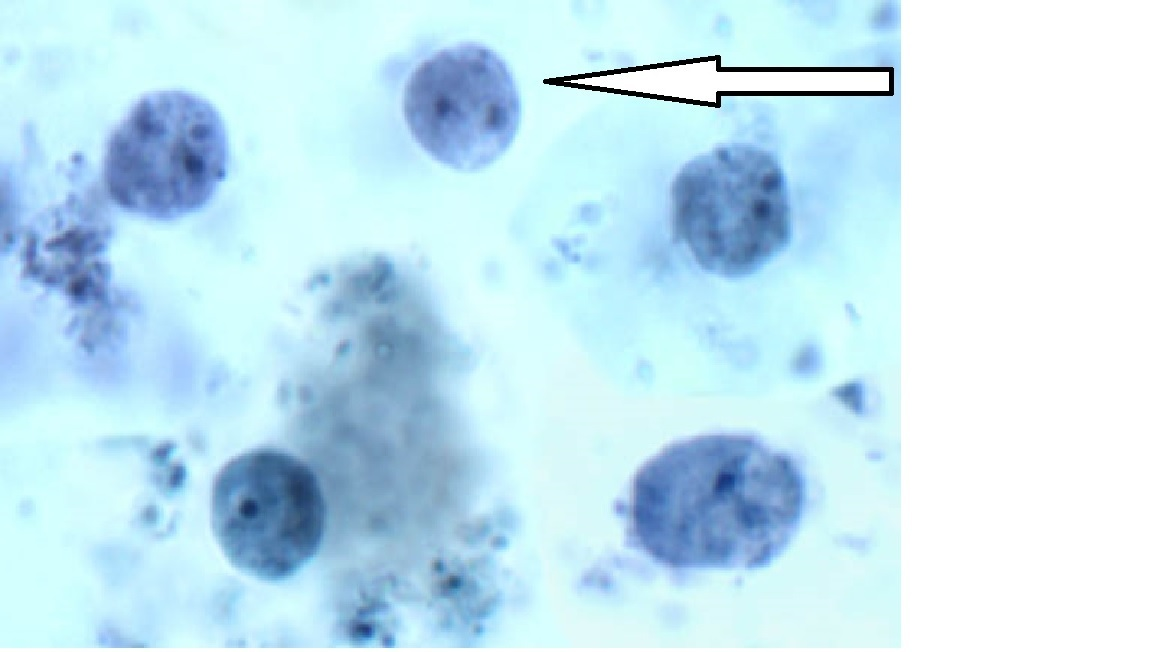
Stool for ova and parasites Dientamoeba fragilis from a stool sample. Trophozoites exhibit an ameba-like morphology and are often bi-nucleated (solid arrow).

A 33-year-old African American male without a significant past history was seen in the office with a three-month history of intermittent, generalized, crampy abdominal pain. It was associated with bloating, cramping, flatulence, and mild diarrhea. The patient reported a worsening with stress and heavy meals and improvement after a bowel movement. He denied any associated weight loss. He reported visiting Mexico for his honeymoon four months ago. Initial lab work, including complete blood count, serum chemistry, inflammatory markers, and serum thyroid stimulating hormone were normal. Dietary changes, including the fermentable oligosaccharides, disaccharides, monosaccharides, and polyols (FODMAP) diet and loperamide were prescribed with the presumptive diagnosis of irritable bowel syndrome. However, the patient’s symptoms persisted. Three samples of stool for ova and parasites (O&P) were ordered, and all of them returned positive for trophozoites of D. fragilis (Figure 1).

The patient was treated with metronidazole for 14 days. Repeat fecal O&P was negative. Upon follow-up four weeks later, the patient's symptoms substantially improved with a resolution of abdominal pain and flatulence.

## Discussion

Parasitic infections are widespread in both urban and rural areas throughout the world. Their prevalence is higher in areas of poor sanitation and drinking water, as they are transmitted via the fecal-oral route [1]. Prevalence rates range between 0% and 52%, depending on the population studied and the method used for detection [8].

D. fragilis is a binucleated protozoan related to Trichomonads; initially classified as amoeba but reclassified as a flagellate, although it lacks a flagellum. Studies have shown that two genetically distinct forms of this organism exist, but their clinical significance is not very well studied [4].

Humans are the preferred hosts, although rodents, pigs, and gorillas are also considered natural hosts. Transmission of D. fragilis is via the fecal-oral route. Trophozoites of D. fragilis are very fragile and degenerate rapidly once passed out of the human body; hence, they are named ‘fragilis’ [9]. In addition, its cyst forms were isolated from the human samples; however, based on the rarity of cysts in those samples, it is difficult to state whether they have any role in disease transmission [9].

Infection caused by D. fragilis may be symptomatic or asymptomatic. Symptoms include abdominal pain, bloating, and alteration of bowel movements, resembling IBS [7]. It was first reported by Australian physicians in 2002. TJ Borody et al. conducted a prospective study where 21 patients presented with IBS-like symptoms for more than a two-month duration, including chronic diarrhea, constipation, abdominal pain, bloating, flatulence, and anorexia. Stool samples were obtained from all patients. All of them were positive for D. fragilis and were treated with antibiotics for 20 days. Out of the 21, 14 patients (67%) experienced resolution of symptoms at four weeks post-eradication [1].

The detection of D. fragilis in stool specimens largely depends on the method of detection employed. Using suitable staining techniques and appropriate culture media and increasing sampling to at least three specimens significantly increases the detection rate. Microscopic identification solely depends on detecting D. fragilis trophozoites in the appropriately fixed and stained fecal specimens. These permanent staining techniques are time-consuming and require highly experienced microscopists to interpret the stains. Parasite culture is the most sensitive method for detecting D. fragilis when directly compared to permanent staining techniques. The culture has a higher yield and is less laborious than staining but, unfortunately, this method is not employed in routine diagnostic laboratories. A deoxyribonucleic acid (DNA)-based diagnosis by a polymerase chain reaction (PCR) is available for the detection of D. fragilis but not widely used [1].

Treatment is warranted when the organism is found as a sole pathogen in stool samples in the setting of abdominal pain or diarrhea lasting more than one week. TJ Boroty et al. reported that 67% of his patients with IBS-like symptoms had successful eradication of D. fragilis, resulting in a complete resolution of symptoms when treated with iodoquinol and doxycycline [7].

## Conclusions

There is an association between a D. fragilis infection and IBS. D. fragilis must be considered as a differential and must be ruled out. When eradicated successfully, D. fragilis-associated IBS results in a complete resolution of symptoms. Further studies are needed to better understand the pathology and pathogenesis of post-infectious IBS caused by D. fragilis.
